# Revisiting DNA Sequence-Dependent Deformability in High-Resolution Structures: Effects of Flanking Base Pairs on Dinucleotide Morphology and Global Chain Configuration

**DOI:** 10.3390/life12050759

**Published:** 2022-05-20

**Authors:** Robert T. Young, Luke Czapla, Zoe O. Wefers, Benjamin M. Cohen, Wilma K. Olson

**Affiliations:** 1 Department of Chemistry & Chemical Biology, Center for Quantitative Biology, Rutgers, The State University of New Jersey, Piscataway, NJ 08854, USA; ryoung.2011@rutgers.edu (R.T.Y.); czaplaluke@gmail.com (L.C.); zoewefers@comcast.net (Z.O.W.); b.cohen479@gmail.com (B.M.C.); 2 Memorial Sloan Kettering Cancer Center, New York, NY 10065, USA

**Keywords:** DNA sequence-dependent structure, DNA deformability, DNA sequence context, DNA curvature, DNA minicircles

## Abstract

DNA carries more than the list of biochemical ingredients that drive the basic functions of living systems. The sequence of base pairs includes a multitude of structural and energetic signals, which determine the degree to which the long, threadlike molecule moves and how it responds to proteins and other molecules that control its processing and govern its packaging. The chemical composition of base pairs directs the spatial disposition and fluctuations of successive residues. The observed arrangements of these moieties in high-resolution protein–DNA crystal structures provide one of the best available estimates of the natural, sequence-dependent structure and deformability of the double-helical molecule. Here, we update the set of knowledge-based elastic potentials designed to describe the observed equilibrium structures and configurational fluctuations of the ten unique base-pair steps. The large number of currently available structures makes it possible to characterize the configurational preferences of the DNA base-pair steps within the context of their immediate neighbors, i.e., tetrameric context. Use of these knowledge-based potentials shows promise in accounting for known effects of sequence in long chain molecules, e.g., the degree of curvature reported in classic gel mobility studies and the recently reported sequence-dependent responses of supercoiled minicircles to nuclease cleavage.

## 1. Introduction

Encoded in the strings of DNA bases that make up the genomes of living species are codes that underlie an assortment of biological processes. The underpinnings of these codes lie in the base sequence-dependent energetic and structural features of DNA, which dictate the degree to which the long, threadlike molecule fluctuates and how it responds to the proteins and other molecules involved in its activity and packaging. The preferred arrangements of base pairs determine the natural folding of individual sequences, as well as the ease with which these folds deform from their equilibrium rest states.

**Figure 1:**
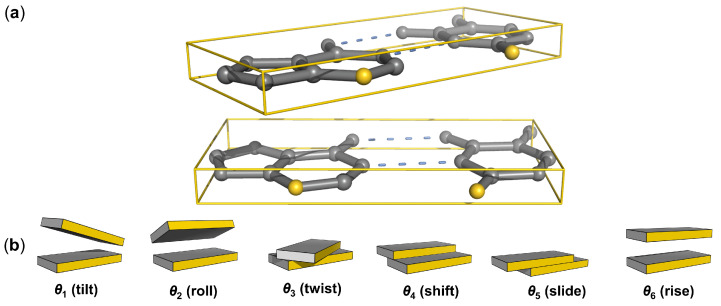
(**a**) All-atom model highlighting, in gold, the purine N3 and pyrimidine O2 atoms on the minor-groove edge of a DNA base-pair step. The orientation and displacement of successive base pairs, enclosed in rectangular slabs, are described in terms of six rigid-body parameters. (**b**) Schematics illustrating positive values of each parameter with the sequence-bearing strand at the left and the minor groove edge (gold) facing the reader.

DNA structure depends upon both the underlying base sequence and the local chemical environment. Variations in the environment introduce large-scale rearrangements of the canonical right-handed double helix with 10 base pairs per turn [1] to under- and overwound structures of the same helical sense with, respectively, more or fewer base pairs per turn [2,3]. These changes in helical state are coupled to changes in the orientation and displacement of successive base pairs and to rearrangements of the intervening sugar-phosphate backbone [4]. Whereas the planes of base pairs pass through and stack roughly perpendicular to the global helical axis of canonical B DNA, they deviate from this alignment in under- and overwound forms. The changes of helical state introduce slight bends between successive base pairs and lateral movements that open channels through the center of the double helix and expose or hide different nucleotide atoms [5]. For example, the underwound A form of DNA is compacted relative to B DNA with atoms on the minor-groove edges of base pairs, i.e., the edges of the bases containing the pyrimidine O2 and the purine N3 atoms (Figure 1), much more exposed to the surrounding chemical environment than in B DNA. By contrast, the major-groove edge, on the opposite side of the base pairs, becomes more accessible in the overwound C form of DNA.

The sequence introduces an even finer level of detail in DNA organization. The base pairs found in high-resolution structures deviate from the ideal, planar hydrogen-bonded arrangements anticipated by Watson and Crick [6] and do not align one above another in perfectly ordered arrays. The chemical composition of the bases directs the spatial disposition of successive residues [7,8], playing a critical role in the overall pathway of the chain and in processes involving DNA recognition. The helix tends to unwind at certain base-pair steps with accompanying changes in bending and displacement of the sort found in A DNA [9]. Other steps exhibit a propensity to overwind and adopt conformational states more characteristic of C DNA [10]. The shifting of base pairs between the different helical forms introduces local kinks in the DNA that have pronounced effects on the overall molecular pathway [11,12].

Analysis of high-resolution DNA structures has revealed subtle sequence-dependent irregularities in the apparent rest state and fluctuations of successive base pairs [13]. The observed correlations in base-pair orientation and displacement in crystalline complexes with proteins have led, in turn, to the determination of a set of knowledge-based elastic energy functions widely used for understanding the nucleic acid machinery. The average values and correlations in observed structural parameters have also provided useful benchmarks for checking state-of-the-art, atomic-level DNA calculations and have stimulated the extraction of similar elastic functions from the features of base pairs in large ensembles of computer-simulated molecules [14]. Compared with the experimental data, the computed datasets include vastly larger numbers of configurational ‘snapshots’ and describe DNA behavior in specific local chemical environments. The analysis of DNA variability within ensembles of protein–DNA structures assumes that different proteins impose different sorts of forces on DNA, that these forces effectively cancel one another (major vs. minor groove bending, etc.), and that the natural conformational response of DNA surfaces after averaging over the dataset [13]. On the other hand, the features of simulated DNA molecules depend upon the number of computed configurations and the reliability of the force fields used to describe atomic-level interactions [15].

The small number of high-resolution structures limited early analyses of DNA sequence-dependent structure and deformability to the dimer level, i.e., the spatial orientation and displacement of successive base pairs. Various properties of DNA—such as the preferred positioning of nucleosomes on DNA [16,17], the cutting patterns of the DNA backbone by hydroxyl radicals [18,19], the mobilities of synthetic oligonucleotides on gels [20], the profiles of DNase I digestion on restriction fragments and phage promoters [21], etc.—depend upon sequence content, i.e., the identities of the base pairs flanking a dimer. Moreover, analyses of simulated DNA structures point to variability in the rest states and deformations of a given dimer within different tetranucleotide environments [22,23,24,25,26,27,28], i.e., the identities of the immediately preceding and following base pairs. The substantial number of high-resolution nucleic acid structures now available makes it possible to examine the corresponding effects in protein–DNA crystal complexes. The data include a much wider variety of DNA-bound proteins than originally examined, with 40-fold or more structural examples of each unique dimer (see results below).

This article starts with an update of the apparent equilibrium rest states and intrinsic deformations of successive base pairs in protein–DNA complexes, highlighting trends in the data accumulated since determination of the first set of knowledge-based potentials. Next follows an overview of the effects of tetrameric context and resolution on the structural features of the base-pair steps in the collected data. The discussion focuses on the twist between successive base pairs and the consequent sequence-dependent under- and overwinding of DNA, as well as on the relative deformability of the base-pair steps. The narrative then turns to an examination of the extent to which the current dimer and tetrameric models of sequence-dependent DNA structure and deformability take account of known effects of sequence on apparent DNA curvature in classic gel mobility studies [20,29,30,31]. The test of the data involves comparison of the reported degree of curvature in selected series of concatenated oligonucleotide sequences with the ring closure propensities of modeled DNA minicircles of identical composition and chain length, i.e., number of base pairs (bp). The paper concludes with predictions of the effect of sequence on the preferred configurations of a well-characterized 336-bp minicircle and the extent to which the updated models take account of known sites of enzyme cutting on the DNA [32].

## 2. Materials and Methods

### 2.1. Dataset

The features of DNA base-pair steps reported herein are based on an ensemble of configurational states found in a collection of 3971 protein–DNA crystal structures extracted from the Protein Data Bank (pdb) [33] in February 2022. The dataset (see Table S1 in Supporting Material) excludes redundant structures, such as those solved independently under slightly different crystallographic conditions, with modifications of a few base pairs, with a mutant protein in place of the wild-type protein, etc. These structures are identified with a new automated procedure, which uses ECOD (evolutionary classifications of domains) identifiers [34,35] reported within each pdb file in combination with DNA sequence matching. The sequences in a pair of structures are taken as matched if the longest stretch of identical base pairs in the two structures is more than 70% of the total DNA length, and pairs of structures are deemed redundant if sequences so matched associate with a protein with the same ECOD identifiers, specifically the same number and types of domains (F-group/H-group names). The structure of better resolution is added to the dataset and the remaining structure is discarded. Duplicate helices in symmetric structures, e.g., two of the three helices comprising a three-armed junction, are also excluded. The resulting, nearly random sample of protein-DNA structures removes bias associated with the repetition of nearly identical structures, thereby allowing for a more uniform exploration of DNA configuration space.

### 2.2. Configurational States

The configurational states of base-pair steps within the selected structures are expressed in terms of the six rigid-body parameters—tilt, roll, twist, shift, slide, rise (Figure 1)—commonly used to describe the relative orientation and displacement of coordinate frames on successive base pairs [36]. The base-pair frames are located on the mid-frame between complementary bases, following the fitting procedure and rotational scheme used in 3DNA [5]. The angular parameters are extracted from the Euler angles used in the rotational scheme and the translational parameters from the vector that connects base-pair origins, when expressed in the mid-frame (see [37] for mathematical details). Numerical values are collected for all base-pair steps in a given structure and placed into 10 unique dimer groups, taking account of the sign differences of tilt and shift in non-unique dimers compared to those on the complementary unique base-pair steps [36]. The step parameters are further classified in terms of tetrameric context, with the 256 possible combinations of four successive base pairs reduced to 136 unique values, i.e., the 16 possible combinations of base pairs flanking each of the six unique non-self-complementary base-pair steps and the 10 possible combinations flanking each of the four self-complementary steps (136 = 16 × 6 + 10 × 4; see Figure S1). Base pairs at the ends of chains or adjacent to ‘melted’ (unpaired) steps are placed into separate groupings, which are not considered here. Each set of tetramers is then subjected to a culling procedure that excludes outlying states of extreme deformation in a stepwise fashion until there are no base-pair step parameters more than three standard deviations from their average values. In practice, quasi-Gaussian distributions of rigid-body parameters are obtained after 3–15 rounds of such culling. Even though the culling is restricted to the six rigid-body parameters, the procedure eliminates almost all non-canonical base pairs, e.g., wobble or Hoogsteen pairs with distinctly different interbase arrangements. The base pairs in the collected set of structures exhibit minor fluctuations about the ideal, Watson–Crick configuration [38], with occasional occurrences (<2% depending upon tetrameric context) of partially melted states with missing hydrogen bonds or slight in- and/or out-of-plane deformations. The six base-pair parameters—so-called buckle, propeller, opening, shear, stretch, stagger [36]—adopt values similar to those previously reported for double-helical structures [39,40]. Small uncertainties in the positions of individual atoms have limited effect on the computed values of both the base-pair and the base-pair-step parameters [41].

### 2.3. Knowledge-Based Potentials

Average step parameters are determined for the 10 unique dimers in different sequence, temporal, and resolution contexts. Dimer averages are determined in two ways—averages of step parameters over all structural examples and weighted averages over all possible tetrameric contexts, i.e., averages of the mean step parameters of the 16 combinations of flanking base pairs. Tetramer averages, i.e., the mean configurational parameters of dimers in a specific tetramer context, are determined over the available structures. Knowledge-based elastic energy functions are generated, as described previously [13], from the mean values and dispersion of the step parameters of dimers and tetramers in the collected data. The deformability of base-pair steps is reported in terms of the average volume of configuration space $\langle V_{\mathrm{step}}\rangle$ accessible to the different steps. Values are determined from the product of the square roots of the eigenvalues of the covariance matrix, i.e., the 6 × 6 matrix with elements corresponding to the differences between the mean products and products of mean values $\langle \Delta \theta_{i}\Delta \theta_{j}\rangle$ = $\langle \Delta \theta_{i}\theta_{j}\rangle$ – $\langle \theta_{i}\rangle \langle \theta_{j}\rangle$ of all combinations of step parameters, where $\theta_{i}$ (*i* = 1–6) refers to one of the rigid-body parameters. Temporal averages are based on datasets collected up to and including a given year. Resolution averages are based on structures at or better than a specified limit.

Properties of a generic MN base-pair step are described in terms of both sequence-dependent averages, obtained by equally weighting the average step parameters of the 16 possible base-pair steps, and structure-based averages, evaluated over sets of available structures. In order to remove bias associated with the unequal number of examples of different base-pair steps in the collected data, the latter averages are based on the same number of examples of each type of dimer step, here 70% of the number of examples of the least represented dimer. The data are collected by randomly sampling a subset of the configurations associated with each of the 10 unique base-pair steps. The configurations of non-unique complementary steps are described by the same structural examples with requisite changes in the signs of tilt and shift ($\theta_{1}$, $\theta_{4}$) [36]. Self-complementary steps include the step parameters of both strands so that the averages values of tilt and shift are null. The MN parameters reflect the combined subsets of configurations, i.e., the set of step parameters and the configurational volumes collected for all 16 base-pair steps. The process is repeated several hundred times in the context of the year in which the data were available and the resolution of the most recently collected data.

### 2.4. Energy Optimization

The sequence-dependent configurations of 150-bp DNA minicircles are obtained using emDNA, new software that optimizes the energy of a collection of base pairs, in which the first and last pairs are held fixed [42,43]. The DNA is described at the level of base-pair steps in terms of the six rigid-body parameters and guided by the knowledge-based potentials described above. The configuration of the DNA as a whole is monitored by a second set of variables that keep track of the vectorial displacements of successive base pairs in a global reference frame. The introduction of the latter quantities makes it possible to take direct account of the spatial constraints imposed on the DNA and to use unconstrained numerical optimization methods. The linking number Lk is controlled by the twist assigned to uniformly spaced base pairs in the initial circular starting structure, here approximated by the rigid-body parameter of the same name (Figure 1) and assigned values based on the expected total twist of a relaxed chain. Supercoiled chains are assigned differences in linking number ΔLk relative to this reference. The total twist, or the total number of turns of helix in the starting structure, is obtained by dividing the sum of the assumed equilibrium twist angles, in degrees, by 360°. The total twist of the optimized structure is measured in terms of the twist of supercoiling, a quantity that takes account of both the rotational and the translational contributions to the wrapping of DNA strands about one another [44,45] as opposed to the step parameter used herein to characterize and build three-dimensional DNA models. The two twists are nearly identical in the optimized structures, where lateral displacements of successive base pairs are minimal. A Debye–Hückel term is used to prevent the self-contact of DNA residues separated by 11 bp or more. The charge on each phosphate group is placed on the base-pair center and assigned a value –0.24 esu in accordance with the predictions of counterion condensation theory [46]. The dielectric medium is taken to be that of a 100 mM aqueous monovalent salt solution.

### 2.5. Ring-Closure Propensities

The simulated ring-closure propensities, or *J*-factors, of short minicircles are compared with reported values of DNA curvature. The ease of cyclization is estimated from the statistical weights of the energy-optimized configurations. This treatment ignores other features of the system that might contribute to the free energy, e.g., base-pair melting, long-range attractive forces, room-temperature fluctuations, etc. DNA curvature is estimated by interpolation of the ratios of the apparent chain lengths of multimer sequences, determined from comparison with size markers on polyacrylamide gels, in published figures [20,29,30,31]. The predicted sequence-dependent uptake of twist in different topoisomers of optimized DNA minicircles is compared with observed hotspots of enzymatic cleavage [32].

## 3. Results

### 3.1. Base-Pair Steps within High-Resolution Structures

**Figure 2:**
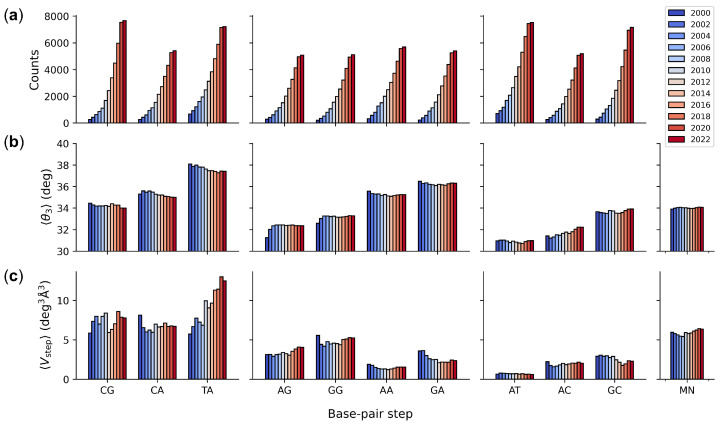
Color-coded histograms illustrating sequence-dependent features of DNA found within high-resolution protein–DNA structures collected over the last two decades: (**a**) number of base-pair steps; (**b**) intrinsic dimer structure measured in terms of the average twist angle $\langle \theta_{3}\rangle$ between successive base pairs; (**c**) dimer deformability measured in terms the average volume of configuration space $\langle V_{\mathrm{step}}\rangle$ accessed by individual steps. MN parameters for a generic MpN step based on equal weighting of the average parameters of the 16 common dimers. Base-pair steps grouped by chemical class (pyrimidine–purine, purine–purine, and purine–pyrimidine). See Table S2 for numerical values and Methods for details.

The original knowledge-based description of DNA sequence-dependent structure and deformability derived from a hand-curated dataset of 92 non-redundant protein-DNA crystal structures with ∼100 examples of each unique base-pair step [13]. There are now in excess of 5000 examples of each step, including more than 7000 examples of each of the four self-complementary steps, in the current collection of computationally curated structures (Figure 2a). The latter counts include steps from both strands of the sampled DNA structures, given that the signs of these parameters differ when expressed in terms of the leading or complementary chain [36]. Although the curation of structures excludes 48 of the 3971 selected protein–DNA complexes, the build-up over time of the number of examples of the 10 unique base-pair steps has been exponential, save for a pandemic-related leveling off of new entries in 2020–2022. The most and least represented dimers in the current collection are CG/CG and AG/CT steps with 7670 and 5078 examples, respectively (see Table S2 for a complete enumeration of structural counts).

Many of the sequence-dependent features of DNA base-pair steps found in the original set of protein–DNA crystal structures have not significantly changed over time. The average twist of individual dimers has levelled off to characteristic values, within ∼2° of the original values (see Table S2 for details). Moreover, the same trends in the relative magnitude of twist deduced from early biophysical studies of DNA in gels and in solution [47] and found in the first few high-resolution structures [8,13] also persist (Figure 2b). That is, the twist of pyrimidine–purine, purine–purine, and purine–pyrimidine steps continues to increase in the same order—CG < CA < TA, AG < GG < AA < GA, and AT < AC < GC, respectively. Moreover, the 34.1° twist of a generic MN dimer, obtained by equally weighting the average twist values of the 16 possible base-pair steps, shows remarkable agreement with the 10.6 base-pair helical repeat of mixed sequence DNA found in pioneering micrococcal nuclease cutting [48] and electrophoresis gel band-shift [49,50] measurements, i.e., 360°/turn÷34.1°/bp = 10.6 bp/turn.

The deformability of base-pair steps, as measured in terms of the average volume of configuration space $\langle V_{\mathrm{step}}\rangle$ enclosing the principal axes of dimeric distortion, has also remained much the same over time, save for appreciable growth in the apparent mobility of TA steps (Figure 2c). The volume occupied by current TA structural examples is roughly double that reported originally and appreciably greater than that of any other base-pair step. Moreover, the range of TA movement does not yet appear to have reached an asymptotic limit with the addition of most recent structural examples. The AT step, by contrast, remains the stiffest dimer in terms of $\langle V_{\mathrm{step}}\rangle$, with less than a 10th of the volume accessible to the TA step and very limited change in magnitude as new structures have accumulated (see Table S2 for numerical details). The plotted deformability of a generic MN step is an average over the configurational volumes of the 16 possible base-pair steps. The resulting values of $\langle V_{\mathrm{step}}\rangle$ have remained relatively constant over time, with magnitudes comparable to those of CA base-pair steps. The structure-based values of $\langle V_{\mathrm{step}}\rangle$, obtained from random subsets of structures available in the database in a specified year (see Methods), are ∼85% of the sequence-based volumes (see Table S2 for comparative values).

**Figure 3:**
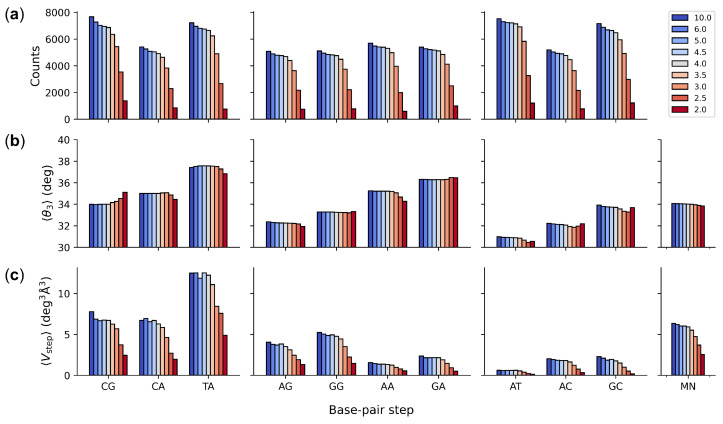
Color-coded histograms illustrating sequence-dependent features of DNA found in high-resolution protein–DNA structures of specified resolution accumulated as of February 2022: (**a**) number of base-pair steps; (**b**) intrinsic dimer structure measured in terms of the average twist angle $\langle \theta_{3}\rangle$ between successive base pairs; (**c**) dimer deformability measured in terms the average volume of configuration space $\langle V_{\mathrm{step}}\rangle$ accessed by individual steps. See Table S3 for numerical values.

Structures of 3.0 Å or better resolution make up roughly two-thirds of the most recently accumulated dataset with mean values of twist not substantially different from those of dimers in the complete set of structures (Figure 3a,b). The average twist of base-pair steps in the ∼15% best resolved structures, with 2 Å or better resolution, show somewhat larger differences in value (as much as 1°) from those of the complete dataset. The variation in the relative magnitudes of twist in the smaller dataset also differs slightly from that noted above, with the twist of CA steps slightly lower than those of CG steps. The deformability of base-pair steps is fairly sensitive to resolution, with individual steps in the 3.0 Å subset of structures occupying ∼60% of the volume accessible to the corresponding dimers in the full dataset (Figure 3c). The accessible volume is even smaller in the set of best-resolved structures (<2 Å), with the volume occupied by the best-resolved GC steps less than 10% of that in the full dataset (see Table S3 for details). The configurational volumes of generic MN steps show similar decreases in value with improved resolution. The structure-derived values of $\langle V_{\mathrm{step}}\rangle$ again fall short of the sequence-averaged values (see Table S3 for numerical comparisons).

### 3.2. Effects of Sequence Context on Base-Pair Structure and Deformability

**Figure 4:**
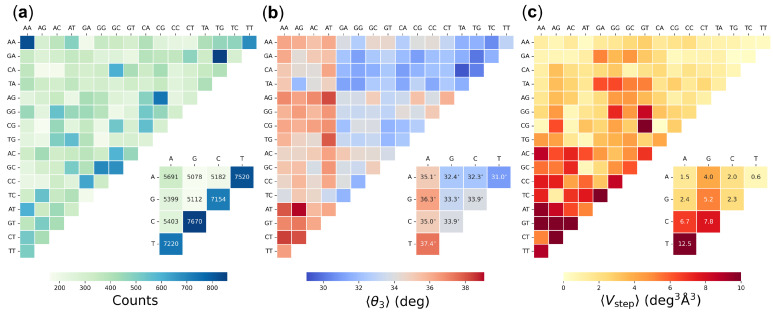
Color-coded heat maps of (**a**) the number of base-pair steps, (**b**) the average twist angle $\langle \theta_{3}\rangle$ between successive base pairs, and (**c**) the average volume of configuration space $\langle V_{\mathrm{step}}\rangle$ accessible to DNA dimers in all unique tetrameric contexts. Data organized such that purine–purine steps lie at the top left of each grid, purine–pyrimidine steps at the lower left, and pyrimidine–purine steps at the top right. Values collected from 3923 high-resolution protein–DNA structures available in February 2022. Dimer values in numbered boxes are averages of the mean values found for each base-pair step in all 16 tetrameric contexts. See Table S4 for numerical values and Methods for details.

The complete set of collected structures includes 160 or more examples of each DNA base-pair step in all possible tetrameric contexts, with a maximum of 869 examples of the configuration of an AT dimer flanked on both sides by a G·C base pair, i.e., the GATG tetramer (see Figure 4a). As anticipated from atomic-level simulations [22,23,24,25,26,27,28], the average structure of successive base pairs is sensitive to the surrounding nucleotide environment. The mean twist angles of a specific base-pair step vary over a range of 3–4° depending upon the identities of the surrounding base pairs. For example, tetramers with a central CG or GC step are equally likely to be slightly under- or overtwisted relative to the 10.6 helical repeat of mixed-sequence DNA, with a nearly equal mix of respective blue and red entries in Figure 4b. The central dimers within other tetramers tend to be either under- or overtwisted relative to the 10.6 reference regardless of the surrounding base pairs, e.g., primarily blue untwisted AG, AC, AT, GG steps vs. largely red overtwisted AA, GA, CA, TA steps. The TAAG step stands out in being highly undertwisted compared to other steps sharing a central AA dimer, with a value of twist characteristic of an 11-fold helix as opposed to the 10-fold structures adopted by the majority of AA dimers (see Table S4 for numerical values). The extremes of twisting occur in AT and TA dimers in the context of CATA and ATAG sequences with average values of 29.3° and 39.5°, respectively. The dimeric averages in the figure inset, which are based on equal weighting of the average twist of each base-pair step in all 16 possible tetrameric context, differ slightly (≤0.1°) from the numbers reported in Figure 2 for the same February 2022 dataset. The latter values are averages evaluated over all structural examples of a given base-pair step, regardless of sequence context.

The collected data also point to examples of dimer deformability influenced by sequence context. Whereas pyrimidine-purine steps are generally much more deformable than purine–purine and purine–pyrimidine steps in terms of spanning a broader range of configuration space, the CA steps within GCAT and TCAG tetramers are surprisingly stiff, with very small values of $\langle V_{\mathrm{step}}\rangle$ compared to other sequence contexts (CA entries, respectively color-coded beige and red/maroon in Figure 4c). A few purine–pyrimidine steps are extremely stiff, with configurational volumes substantially smaller than those of other steps sharing the same central base pairs. For example, the replacement of thymine by cytosine in TGCA compared to CGCA introduces a 30-fold decrease in $\langle V_{\mathrm{step}}\rangle$ (neighboring tan vs. pale beige GC entries in Figure 4c corresponding to the greatest and least deformable GC steps). Among purine–purine steps, AG and GG dimers show greater sensitivity to sequence context than AA and GA dimers, which are nearly as stiff as purine–pyrimidine dimers. The context-averaged dimeric values of $\langle V_{\mathrm{step}}\rangle$ reported in the figure inset show similar trends in magnitude but appreciable numerical differences from the structure-averaged configurational volumes reported in Table S2, particularly in the highly deformable TA and CG steps (see Table S4 for numerical values). The numerical differences are unsurprising given both the various averages contributing to $\langle V_{\mathrm{step}}\rangle$ (see Methods) and the different number of examples of each tetrameric context. The structure-based dimer averages in Table S2 do not consider these differences.

Sequence context has much the same effect on base-pair step configuration and deformability in the subset of recently collected structures with 3.0 Å or better resolution. The latter data include 91 or more examples of each base-pair step in all tetrameric contexts, with 789 examples of the AT dimers within a GATG tetramer (the same sequence context found in greatest number in the complete set of recent structures regardless of resolution). The least populated tetrameric sequence, CGAG, is also common to both sets of structures. Although numerical values of average twist differ in individual cases, the trends in relative magnitude persist. For example, AT and TA dimers with respective twists of 28.4° in CATA tetramers and 39.9° in ATAG tetramers remain at the extremes of under- and overtwisting relative to the same 10.6 helical repeat of mixed-sequence DNA. The differences in average twist between the smaller and larger datasets range between –1.4° and +1.9° with an average magnitude of 0.3°. The trends in relative dimer deformability also persist despite the average 60% drop in $\langle V_{\mathrm{step}}\rangle$ over all tetrameric contexts. The base-pair steps within some tetramers show little change in deformability between the two datasets (e.g., CTAA, TCGA, ACGT, TAGT) whereas others show very substantial drops in configurational volume (e.g., more than an order of magnitude decrease in CA deformability in the very stiff GCAT sequence noted above). See Table S5 for numerical details.

### 3.3. Sequence-Dependent DNA Curvature

**Figure 5:**
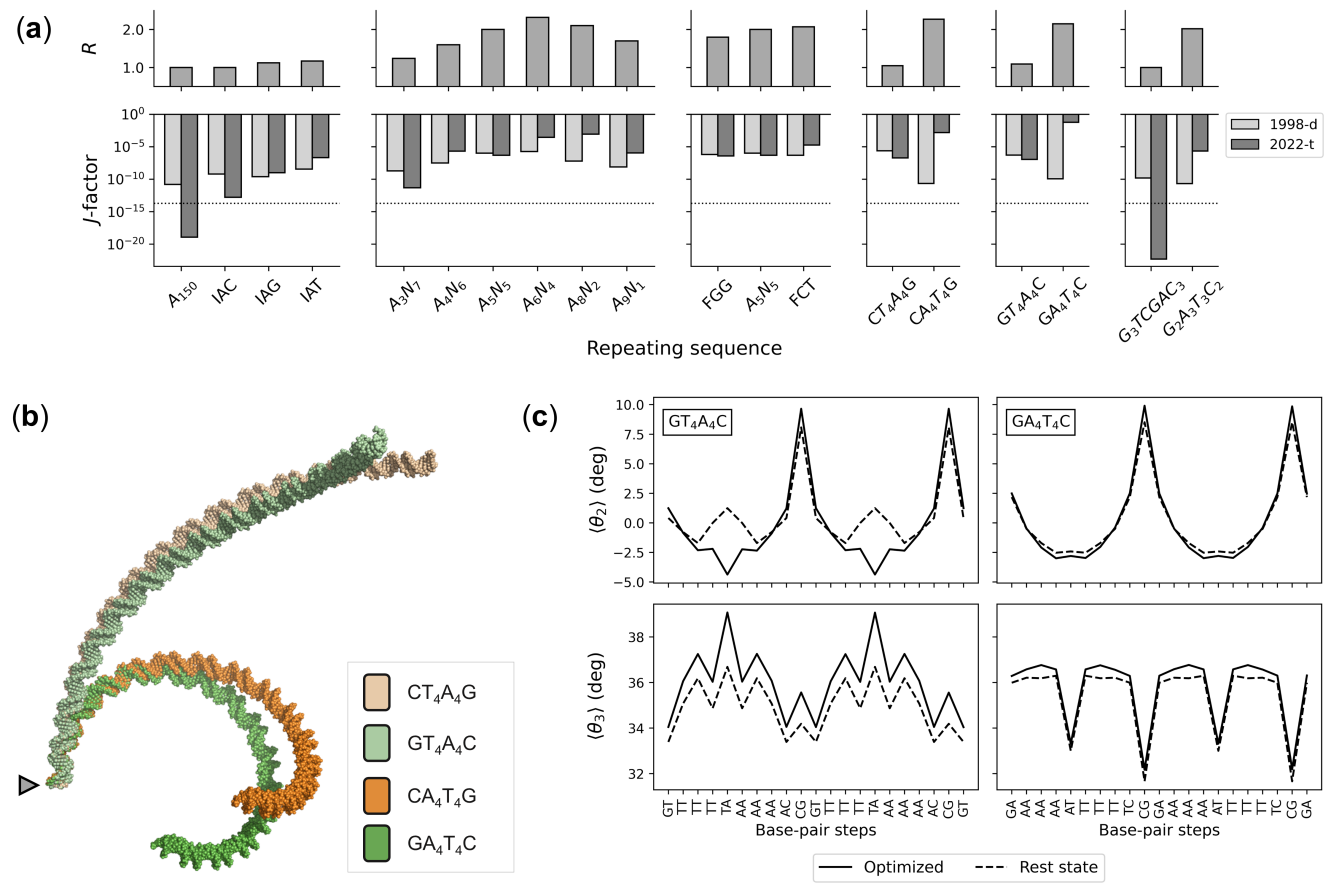
Sequence-dependent estimates of DNA ring closure compared with the degree of curvature determined in classic gel electrophoresis studies [20,29,30,31]. (**a**) Variation in DNA curvature, measured in terms of the ratio *R* of the apparent chain length to the true chain length, and in the computed *J*-factors of covalently closed 150-bp polymers bearing various 10-bp repeating sequences. *J*-factors extracted from the energies of minicircles optimized with the specified knowledge-based potentials: 1998-d (dimeric model reported in [13]); 2022-t (updated model that takes account of tetrameric context); ⋯⋯⋯ (ideal DNA). (**b**) Superimposed molecular images of the intrinsically straight and curved pathways of chains with N$T_{4}A_{4}$N and N$A_{4}T_{4}$N repeating sequences, where N is G or C, found with the tetrameric model. Pathways described in the frame of the first base pair (triangle). (**c**) Concerted changes in local DNA structure that underlie the predicted *J*-factors of the G$T_{4}A_{4}$C and G$A_{4}T_{4}$C sequences. Average values of roll 〈$\theta_{2}$〉 and twist 〈$\theta_{3}$〉 of dimer steps along a 21-bp stretch of the intrinsic linear structures are compared with those on the 150-bp minicircles optimized with the tetrameric model. Trends are similar for C$T_{4}A_{4}$G and C$A_{4}T_{4}$G sequences. See Table S6 for clarification of sequence acronyms and numerical data.

The sequence-dependent structure and deformability of successive base pairs underlie larger-scale features of double-helical DNA, such as the intrinsic curvature associated with repeated tracts of A·T pairs separated by segments of G+C-rich DNA [51]. Estimates of DNA ring closure guided by the current set of knowledge-based potentials show remarkable agreement with the apparent curvature of assorted sequences determined in classic gel mobility studies (Figure 5a). The predicted cyclization propensities of a collection of 150-bp sequences increase in the same order as the reported ratios of apparent molecular size, based on observed electrophoresis markers of chain length, to the actual chain length [20,29,30,31]. The more easily closed sequences with larger *J*-factors match the more strongly curved sequences with larger apparent molecular sizes. Moreover, the potentials take account of the influence of the A-tract repeating length ($A_{j}N_{10-j}$, where N is G or C), the relative effects of specific bases within or at the ends of A tracts, and the contribution of A-tract polarity to the observed degree of curvature (see Figure 5a and Table S6 for details of sequence acronyms and numerical values). The estimated *J*-factors of energy-optimized circles of 147- and 168-bp chains with an $A_{6}N_{4}A_{6}N_{5}$ repeat, however, exceed experimentally measured values ($∼1\times 10^{-4}$ M) [20], with the treatment incorporating tetrameric context yielding higher ring-closure propensities ($∼1\times 10^{-1}$ M) than those based on dimer structure and deformability alone ($∼6\times 10^{-2}$ M). The optimization procedure used here does not consider fluctuations in DNA structure, which may contribute to the free energy of cyclization.

The updated set of knowledge-based potentials takes correct account of gel mobility data missed with the original set of potentials (labeled 1998-d in Figure 5a). For example, optimized minicircles with $N_{5-j}A_{j}T_{j}N_{5-j}$ repeating sequences now appear to be more curved than those made up of $N_{5-j}T_{j}A_{j}N_{5-j}$ sequences with higher computed *J*-factors. As noted above, the high-resolution structures of protein-bound DNA accumulated as of February 2022 show distinct differences in the preferred arrangements and deformations of AT vs. TA base-pair steps, with the former steps stiff and overtwisted and the latter steps highly deformable and undertwisted. The changes in DNA twisting occur in concert with well-known changes in the bending and lateral displacement of successive base pairs via roll and slide, respectively [52]. The stiffness of the AT steps restricts the slide to negative values (–0.7${}_{\pm 0.1}$ Å) regardless of sequence context but allows for subtle, context-dependent variation in the sign of average roll. The TA steps, by contrast, vary widely, with the roll values spanning roughly twice the range of states sampled by the AT steps and the slide more likely to adopt mean positive values in most tetrameric contexts. The linear equilibrium structures of $N_{5-j}A_{j}T_{j}N_{5-j}$ sequences are both more curved than those of $N_{5-j}T_{j}A_{j}N_{5-j}$ sequences and more easily closed into circular configurations with the updated potentials (see the molecular images and changes in step-parameters that effect ring closure in Figure 5b,c). Whereas the intrinsic step parameters of the rest states in the former sequences closely match those in the circular structures, the base-pair steps along the latter sequence must overtwist and the five steps within the TTTAAA stretch must adopt more negative values of roll and/or slide in order to bring the chain ends into perfect register. This mechanical description of DNA curvature differs from conventional interpretations, which focus on static structural features that might underlie experimental observations—e.g., hypothesized wedges between successive A·T base pairs [53,54], differences in overall helical structure between A tracts and intervening G+C-rich linker segments [20,31], compensatory directions of bending at AT vs. TA steps [55,56,57]. Detailed maps of the collective patterns of base-pair structure and deformability will be presented elsewhere.

### 3.4. Sequence-Dependent Twist Uptake in DNA Minicircles

**Figure 6:**
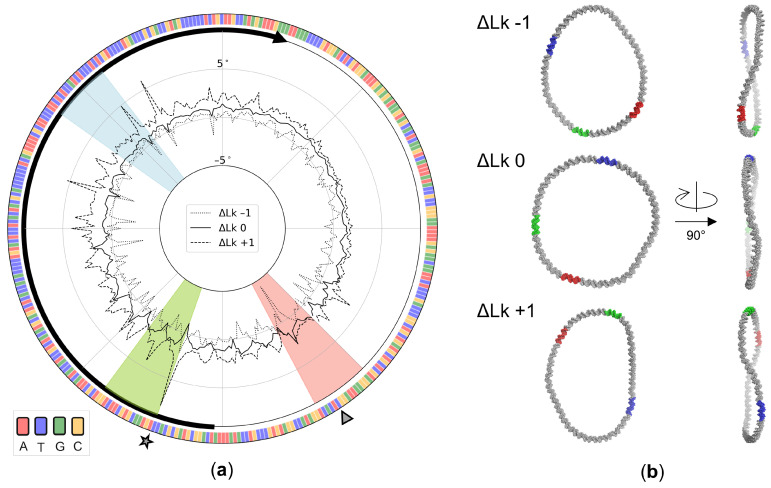
Effects of DNA supercoiling on the optimized configurations of a designed, 336-bp DNA minicircle. (**a**) Build-up of twist, $\Delta \theta_{3}$, in degrees, at individual base-pair steps along pathways optimized with the tetrameric model. The color-coded interior sectors correspond to reported hotspots of enzymatic cleavage [32] and the exterior triangle and star to sites of extreme twist uptake in the optimized structures. The heavy black curve highlights the 180-bp *att*R region that is a remnant of the λ integrase-mediated recombination process used to generate the minicircle [61], with the arrow denoting the $5^{\prime }$–$3^{\prime }$ direction of the chain. The DNA base-pair sequence is color-coded along the outer edge of the figure. (**b**) Atomic-level representations of each optimized minicircle illustrating the changes in overall global shape and the relative locations of the hotspots in the frame of the two longest principal axes (*left*) and the frame of the longest and shortest axes (*right*).

The updated potentials also provide a rationalization behind the sequence-dependent response of designed 336-bp DNA minicircles to nucleases known to cleave segments of ‘melted’ DNA [32,58]. The torsional stress associated with ring closure builds up non-uniformly in structures optimized on the basis of the context-dependent potentials, even in the most relaxed state where the linking number is 32, the total twist is 31.8 helical turns, and ΔLk is taken as zero. Although the twisting at individual base-pair steps deviates very slightly on average (–0.04°) from the intrinsic values along this pathway, the DNA over- and undertwists substantially at selected base-pair steps (see the computed variation in local twist in Figure 6a). Indeed, the twist changes by as much as ±2° and the elastic energy jumps sharply at two TA steps, found in the context of CTAT and TTAC tetramers located, respectively, at residues 144–147 and 193–196 along the published sequence. Moreover, these sites absorb a disproportionately large degree of the twist introduced in the minicircle upon supercoiling. The highly overtwisted TA step within the CTAT tetramer on the relaxed topoisomer becomes substantially undertwisted in the ΔLk = –1 topoisomer while the highly undertwisted step within the TTAC tetramer on the relaxed topoisomer becomes substantially overtwisted in the ΔLk = +1 topoisomer (note the differences in the signs of $\Delta \theta_{3}$ at the sites marked, respectively, by a triangle and a star in the figure). The former step lies within the hotspot for Bal-31 endonuclease cleavage found in negatively supercoiled minicircles, while the latter step abuts the $5^{\prime }$-end of the strong S1 nuclease cleavage site in the same topoisomers [32,58]. Both steps are substantially higher in elastic energy than other steps along the modeled structures with deformation scores 7–10 times larger than the average values. The high energies point to sites of likely helical deformation and the different nature of the torsionally stressed steps to different modes of DNA distortion. Although the two enzymes have well-proven utility for probing disruptions in double-helical DNA [59,60], the precise details of protein–DNA recognition remain unknown. The predicted sites of localized overtwisting likely convert to different distorted forms from the predicted sites of undertwisting. The current potential functions do not take account of distortions within individual base pairs and associated movements of the sugar-phosphate backbone that ‘melt’ double-helical DNA.

The small changes in ΔLk have limited effect on the overall fold of the optimized minicircles. The optimized supercoiled structures adopt more elongated pathways with greater out-of-plane bending than the relaxed structure (Figure 6b). Although the configurations appear to be open from most perspectives, distant segments of the minicircles cross above or below one another in some viewpoints. Moreover, the relative locations of the enzymatic hotspots change with respect to the global features of the structure. The illustrated examples, depicted with two of the three principal axes of each configuration running along the horizontal and vertical directions of the page (axes 1, 2 in the left images and 1, 3 in the right), reveal the out-of-plane character of the supercoiled configurations compared to the relaxed ΔLk = 0 state, as well as the different directions of chain crossing in the positively vs. negatively supercoiled states (Figure 6b, *right*). The enzymatic hotspots rotate in a clockwise direction in the ΔLk + 1 topoisomer and a counterclockwise direction in the ΔLk − 1 topoisomer relative to the positions found for the relaxed state (Figure 6b, *left*).

## 4. Discussion

DNA base sequence carries a multitude of structural and energetic signals important to its biological activity and organization. Primary sequences of nucleic acid bases describe real three-dimensional structures with individual residues adopting characteristic spatial forms and macromolecular features that reflect the natural rest states and deformations of those structures. The first estimates of DNA intrinsic structure and deformability, extracted nearly 25 years ago from the fluctuations and correlations of the arrangements of successive base pairs within a small set of protein–DNA crystal structures [13], have provided useful insights into the role of sequence in DNA recognition and folding. As illustrated herein, the thousands of structures of protein–DNA complexes accumulated since then exhibit many of the sequence-dependent features of DNA base-pair steps found in the original 92 structures, including the same trends in the values and relative magnitudes of the twist angle between successive base pairs and the relative deformability of base-pair steps. The five remaining base-pair-step parameters either vary over broad ranges tightly coupled to the changes in twist, e.g., roll and slide [52], or exhibit very limited deformations within the set of structures, e.g., tilt, shift, and rise [62].

Although the current study does not consider whether the observed sequence-dependent propensities entail rearrangement of the intervening sugar-phosphate backbones, there are some striking similarities between the under- and overtwisted dimer steps reported here and the backbones found to connect the corresponding bases in a recent survey of high-resolution DNA structures [63]. For example, the CpC and GpG halves of the undertwisted GG dimer show higher than expected tendencies to adopt A-like conformational pathways while the backbones linking the overtwisted CA and TA steps show strong propensities to adopt the BII form characteristic of C DNA. How the sequence-dependent deformability of base-pair steps might be tied to the backbone linkages remains an open question.

The large dataset of currently available structures makes it possible to characterize the conformational preferences of the DNA base-pair steps within the context of their immediate neighbors, i.e., in the context of tetramers, for which there are now hundreds of structural examples of each of the 136 unique tetrameric settings. These data provide critical benchmarks for atomic-level simulations of double-helical DNA, as well as information potentially useful in interpreting the properties of specific DNA sequences. For example, the reported effects of sequence context on relative twist angles extracted from atomic-level simulations of short B DNA fragments [25,27] differ from those found here in high-resolution protein–DNA structures. The general trends in dimer deformability, as measured by the volume of accessible configuration space, show notable similarity, e.g., AT steps are the stiffest and YR steps the most flexible [25]. The configuration space sampled in the simulations, however, exceeds that found in the current set of crystal structures. The predicted effects of sequence on local DNA structure and deformability also depend on the simulation method, e.g., choice of force field [15].

The set of knowledge-based potentials extracted from the mean values and dispersion of base-pair-step parameters in the updated set of protein–DNA structures shows promise in accounting for known effects of sequence in long chain molecules. Estimates of DNA ring closure guided by the current set of potentials closely mirror the degree of curvature reported in classic gel mobility studies [20,29,30,31], including the effects of sequence polarity on curvature that the early set of structure-based potentials failed to match. The updated potentials also provide a rationalization behind the observed sequence-dependent response of designed DNA minicircles to nuclease cleavage [32,58]. The extreme build-up of twist in specific tetrameric contexts along supercoiled models occurs in the vicinity of observed hotspots of enzymatic cleavage. These torsionally stressed structures provide a useful starting point for studies of the ‘melting’ of the double helix necessary for generating the denatured forms of DNA—e.g., extrahelical bases, short single-stranded bubbles—thought to be recognized by the enzymes [58]. The data also provide a useful bridge between the atomic details of the molecular dynamics simulations [58] and the coarse-grained treatment of DNA [64] previously used to model the disruptions of base pairs and localized denaturation in the designed minicircle, revealing structural standards against which the former studies can be checked and identifying features in the observed base associations for improvement of the coarse-grained force field.

## Data Availability

Data available upon reasonable request.

## Supplementary Materials

The following supporting information can be downloaded at: https://www.mdpi.com/article/10.3390/life12050759/s1. This includes a .pdf document containing descriptions of the Supplemental Materials and Supplemental Figure S1 and a .xls file containing Supplemental Tables S1–S6 found on separate worksheets.
