# Supplementary material for: Revisiting DNA Sequence-Dependent Deformability in High-Resolution Structures: Effects of Flanking Base Pairs on Dinucleotide Morphology and Global Chain Configuration
Source: Life (Basel). 2022 May 20;12(5):759. doi: 10.3390/life12050759 (PMC9146901; doi:10.3390/life12050759)
Supplement: Supplementary file 1 [file life-12-00759-s001.zip › life-1686066-supplementary.pdf]

Supplemental Materials

Revisiting DNA Sequence-dependent Deformability in High-resolution Structures: Effects of Flanking Base Pairs on Dinucleotide Morphology and Global Chain Configuration

R.T. Young[1], L. Czapla[1,2], Z.O. Wefers[1], B.M. Cohen[1] and W.K. Olson[1*]

[1] *Department of Chemistry & Chemical Biology, Center for Quantitative Biology, Rutgers, the State University of New Jersey, Piscataway, New Jersey 08854, USA*

[2] *Memorial Sloan Kettering Cancer Center, New York City, New York 10065, USA*

*** Correspondence to Wilma K. Olson (**wilma.olson@rutgers.edu).

Overview

The contents of the Supplemental Materials include a figure illustrating the organization of DNA tetramer sequences used in this work (Figure S1); a table listing all protein-DNA structures collected for this study (Table S1); tables with the counts, twist, and volume features of the 10 unique DNA with respect to year (Table S2) and resolution (Table S3); tables with the counts, twist, and volume features of all 136 unique tetramers and their associated dimers for the entire dataset (Table S4) and for all structures at 3.0-Å or better resolution (Table S5); a table detailing sequence acronyms used in the text and the previously-reported ratios of apparent to real chain length (Table S6). All tables can be found in the accompanying .xls spreadsheet document with each table in a separate worksheet.

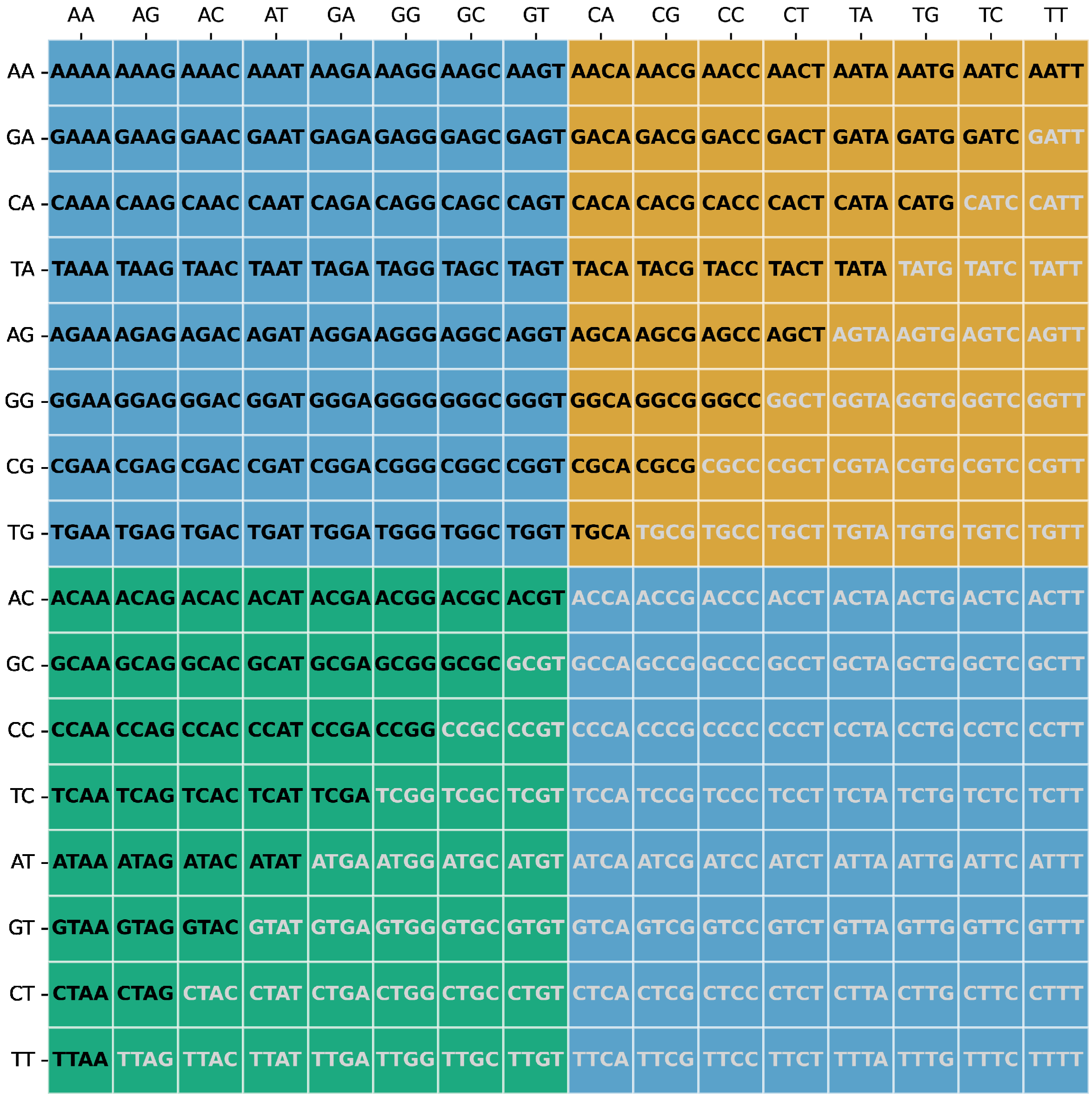

Figure S1. Organization of DNA tetramer sequences used in the current work. Entries above the diagonal, depicted in black font, correspond to the 136 unique tetramers, and those below the diagonal, shown in white font, to the complementary sequences. Data are organized such that purine-purine steps lie at the top left of the grid (blue), purine-pyrimidine steps at the lower left (green), pyrimidine-purine steps at the top right (gold), and pyrimidine-pyrimidine steps at the lower right (blue). Labels along the vertical axis, corresponding to the first two bases, are combined with those along the top horizontal axis, corresponding to the last two base pairs, to generate the listed sequences.
